# Algorithm for Key Transparency with Transparent Logs

**DOI:** 10.12688/openreseurope.18200.1

**Published:** 2024-08-05

**Authors:** Elissa Mollakuqe, Shasivar Rexhepi, Ridvan Bunjaku, Hasan Dag, Ikechukwu John Chukwu

**Affiliations:** 1 Department of Management Information Systems, Kadir Has University, Istanbul, Turkey; 2 Faculty of Computer Sciences, AAB College, Pristina, 10000, Kosovo (Serbia and Montenegro)

**Keywords:** Key, transparency, management, key and public

## Abstract

**Background:**

Cryptography plays a crucial role in securing digital communications and data storage. This study evaluates the Transparent Key Management Algorithm utilizing Merkle trees, focusing on its performance and security effectiveness in cryptographic key handling.

**Methods:**

The research employs simulated experiments to systematically measure and analyze key operational metrics such as insertion and verification times. Synthetic datasets are used to mimic diverse operational conditions, ensuring rigorous evaluation under varying workloads and security threats. Implementation is carried out using R programming, integrating cryptographic functions and Merkle tree structures for integrity verification.

**Results:**

Performance analysis reveals efficient insertion and verification times under normal conditions, essential for operational workflows. Security evaluations demonstrate the algorithm's robustness against tampering, with approximately 95% of keys verified successfully and effective detection of unauthorized modifications. Simulated attack scenarios underscore its resilience in mitigating security threats.

**Conclusions:**

The Transparent Key Management Algorithm, enhanced by Merkle trees and cryptographic hashing techniques, proves effective in ensuring data integrity, security, and operational efficiency. Recommendations include continuous monitoring and adaptive algorithms to bolster resilience against evolving cybersecurity challenges, promoting trust and reliability in cryptographic operations.

## Introduction

Cryptography, the art and science of secure communication, has been a cornerstone of information security for centuries. From ancient techniques of secret writing to modern-day algorithms that protect sensitive digital transactions, cryptography plays a pivotal role in ensuring the confidentiality, integrity, and authenticity of data in communication and storage. The secure management of cryptographic keys is fundamental to ensuring data integrity, confidentiality, and authenticity. Traditional approaches to key management often face challenges related to transparency, scalability, and auditability. The Transparent Key Management Algorithm, leveraging Merkle trees, emerges as a promising solution to these challenges by providing a structured, verifiable framework for cryptographic key management within digital ecosystems. The motivation behind this study lies in the critical need to evaluate and enhance the performance and security of the Transparent Key Management Algorithm in practical applications. As cyber-threats evolve, robust cryptographic key management practices become increasingly vital to mitigate risks such as data breaches, unauthorized access, and tampering. By conducting a systematic evaluation using simulated experiments and rigorous analysis, this study aims to validate the algorithm's effectiveness under diverse operational conditions and security scenarios. Our paper is divided in several sections: in section one we review existing literature and research related to cryptographic key management, with a focus on methodologies utilizing Merkle trees and similar algorithms. We discuss key studies, methodologies, and findings that inform our research on the Transparent Key Management Algorithm. In section two we give details of the methodology adopted for evaluating the Transparent Key Management Algorithm using Merkle trees. It includes sections on data collection methods, simulated experimentation setup, and the overall approach to performance and security evaluation. By including Implementation of Transparent Key Management Algorithm in R, we describe the practical implementation of the algorithm. This section covers the design and development of key functional modules, including key generation, insertion, verification, and auditing methods within the Merkle tree framework. Then we continue with the results section which presents the findings from our study's experiments and analyses. It includes quantitative and qualitative data on algorithmic performance metrics (insertion time, verification time, and computational efficiency) and security evaluations (resilience against tampering, and integrity verification). This section provides a detailed analysis of performance metrics measured during the study. It discusses how R programming was utilized to analyze insertion time, verification time, and computational efficiency under varying operational conditions and key volumes. At the same time, we analyze the security aspects of the Transparent Key Management Algorithm using R scripts. This section examines the algorithm's resilience against simulated attacks, integrity verification mechanisms, and overall robustness in safeguarding cryptographic key management operations. At the end we present Discussion and Conclusion where we interpret the results in the context of our research objectives. This part explores the implications of findings, compares them with existing literature, discusses limitations, and proposes future research directions. The Conclusion summarizes the key findings of the study and highlights the contributions to advancing cryptographic protocols and enhancing data security frameworks.

## Related work

The limitations of alternatives in 2014 have led industry such as Google to develop a new concept called Certificate Transparency, which is a public, verifiable and append-only log for certificates. This log allows third-parties to verify certificates and servers, thus enabling monitoring of misused ones and ensuring that mis-issued certificates are detectable ^1^. In 2015, CONIKS was a key verification system that ensured consistency and privacy for bindings of name-to-key of users without requiring explicit key management by users. This enabled clients to efficiently monitor their bindings and detect equivocation with high probability ^2^. In 2018, supply chain transparency became essential in various industries, being crucial for tracking and addressing issues with goods, such as contamination or damage. Modern concerns about human health, working conditions, and social responsibility have increased the importance of transparency. Environmental, technological, social, health, safety, and ethical considerations started to directly impact business success, highlighting the need for a transparent supply chain ^3^. On 2023 research has evolved and matured on the subject. Traditional approaches like PGP's Web of Trust or Signal's Safety Number did not scale well for millions of users, therefore Key Transparency emerged as a concept that addresses this by making server actions transparent through Merkle hash tree that creates a verifiable key directory, allowing clients to verify the consistency and inclusion of their keys ^4^. Key Transparency systems allow end-to-end encrypted communication platforms to maintain a Verifiable Key Directory that maps user identifiers to their public keys, enabling users to detect attempts at key manipulation ^5^. Encryption alone is not sufficient for secure end-to-end encrypted messaging; the server must also honestly serve public keys to users. Key transparency is an efficient solution for detecting dis-honest servers, involving two main components: a username-to-public key mapping stored and cryptographically committed by the server, and an out-of-band consistency protocol for serving short commitments to users ^6^. As systems more and more impact people's lives significantly, transparency is suggested to identify flaws, enable accountability, and ensure flaws are corrected and mitigated. Transparency is complex and does not specify how it should be implemented, especially in environments hostile to accountability. Questions arise about what information is revealed, in what form, by whom, to whom, and how, and this leads to transparency sometimes being ineffective or counterproductive ^7^. Key Transparency systems ensure the consistent reporting of each user's public key, but prior work has often neglected scalability. Building a scalable and practical KT system is challenging due to complexity, inefficiency, and lack of resilience against machine failures in existing constructions ^8^. In 2024, security and trustworthiness are paramount, achieved through end-to-end encryption and open-source applications for transparency and scrutiny. Public keys, essential for encrypting data before sending them, are retrieved via a key server based on the recipient's username. This process is vulnerable to sophisticated attacks like Man-in-the-Middle, where incorrect public keys could be intercepted and used maliciously. Address Verification is initially used to mitigate this risk, requiring manual verification of keys out-of-and. To improve upon this manual process, it was developed Key Transparency (KT), automating key verification in the background for all users. KT ensures that only legitimate keys are logged and warns users of any discrepancies, providing a similar security level as Address Verification without the need for manual key comparison. Inspired by Certificate Transparency (CT), a Merkle hash tree system is leveraged and integrated with the CT ecosystem for enhanced trust ^9^. WhatsApp’s key transparency system enhances end-to-end encryption by automating key verification. Each device has unique encryption keys, and a compromise on one doesn’t affect others. The system uses Blake3 hash functions and Merkle prefix trees to manage public key directories securely. When a user initiates a conversation, the system provides the recipient’s public key along with a cryptographic proof tied to a global hash digest. This ensures that keys are recorded accurately. Key transparency facilitates device registration, key lookup, and auditing, enabling secure, verifiable communication. Future updates will support key history requests, further bolstering transparency and security ^10^.

## Methods

The study employs a **quantitative** approach to evaluate the performance and security of the Transparent Key Management Algorithm using Merkle trees. This approach allows for systematic measurement and analysis of key management processes, ensuring rigorous assessment of algorithmic effectiveness and resilience. The research design involves simulated experiments to test the Transparent Key Management Algorithm in controlled environments. Synthetic data sets are generated to mimic real-world scenarios, ensuring comprehensive evaluation under various conditions. The methodology adopted for evaluating the Transparent Key Management Algorithm with Merkle trees is comprehensive and systematic, focusing on a blend of quantitative analysis and simulated experimentation. Encompassing a detailed data collection approach, the study leverages simulated data from realistic scenarios to mimic diverse operational conditions and security threats, ensuring controlled testing and robust validation of the algorithm's performance under varying conditions. By prioritizing simulated data for its flexibility and risk mitigation benefits, the research design emphasizes the importance of reproducibility and scalability in generating reliable findings. The study places significant emphasis on key performance metrics such as insertion time, verification time, and computational efficiency to gauge the operational effectiveness of the algorithm. Through meticulous measurement and analysis using R programming, the research team ensures accurate timing evaluations and systematic assessments under different operational contexts and key volumes. Parallelly, the security evaluation component intricately examines the algorithm's resilience against tampering, integrity verification, and unauthorized changes within the Merkle tree structure. Through simulated attacks and monitoring mechanisms facilitated by R scripts, the study validates the algorithm's security capabilities and its ability to withstand evolving cyber threats. The implementation of the Transparent Key Management Algorithm in R programming showcases a meticulous design process that integrates key processes like generation, publication, verification, and auditing methods within the Merkle tree framework. The utilization of robust cryptographic functions like SHA-256 underscores the commitment to data security and integrity, ensuring that each key is securely generated and seamlessly integrated within the hierarchical structure. The integration of encryption techniques, access controls, and audit trails in the R-based implementation further strengthens the algorithm's defense mechanisms against potential vulnerabilities, providing a solid foundation for safeguarding cryptographic key management operations in digital ecosystems. The development of key functional modules within the implemented R environment, such as the functions for generating initial key logs, appending new keys, and analyzing key transparency, facilitates a cohesive framework for data management and analysis tasks. By maintaining a rigorous approach to experimental setup, encompassing comprehensive data generation, in-depth performance metrics analysis, and thorough security evaluation, the study aims to not only validate the Transparent Key Management Algorithm's capabilities but also contribute substantially to the advancement of cryptographic protocols and the fortification of data security frameworks in digital realms.

### Data collection

Based on the nature of our study focusing on cryptographic key management, we have chosen to employ simulated data from realistic scenarios as our primary method for data collection. This approach offers significant advantages in terms of controlled experimentation, reproducibility, and scalability in generating data that simulates diverse operational conditions and security threats. By using simulated data, we can rigorously test and validate the Transparent Key Management Algorithm under various hypothetical scenarios without impacting real operational systems. Simulated data allow us to conduct thorough performance and security evaluations in a controlled environment, ensuring that our research findings are reliable and reproducible. This method also mitigates potential risks associated with accessing and handling sensitive operational data from actual systems, addressing concerns related to data privacy and security compliance. While we prioritize simulated data for its flexibility and controlled experimentation benefits, we remain open to the possibility of collaborating with organizations to access real operational data. Such collaboration could provide authentic insights into actual key management practices, enhancing the realism and applicability of our study's findings. If feasible, combining simulated scenarios with real-world data validation would further strengthen the robustness of our research outcomes, ensuring they are grounded in both theoretical simulations and practical operational insights.

### Data analysis

In evaluating the Transparent Key Management Algorithm using Merkle trees, our study adopts a comprehensive approach centered on rigorous data analysis, performance metrics, and security evaluation. ***Performance metrics*** such as insertion time, verification time, and computational efficiency are crucial benchmarks used to gauge the algorithm's operational effectiveness. Using R programming, we meticulously measure insertion time by capturing the duration for adding new keys into the Merkle tree, ensuring that each operation is timed accurately using system time functions. Verification time, equally vital, assesses the algorithm's speed and reliability in verifying key authenticity within the tree structure, employing systematic timing evaluations to validate performance under varying operational conditions and key volumes. Computational efficiency, a cornerstone in algorithm assessment, is rigorously analyzed through R's statistical tools ( https://posit.co/download/rstudio-desktop/), scrutinizing CPU usage, memory allocation, and scalability to ensure optimal performance across different workloads. In parallel, our study places a significant emphasis on security evaluation, examining the algorithm's resilience against tampering, integrity verification, and detection of unauthorized changes within the Merkle tree. Resistance to tampering is tested through simulated attacks in R, challenging the algorithm's ability to withstand unauthorized modifications through robust cryptographic mechanisms like SHA-256 hashing. Integrity verification processes traverse the Merkle tree structure using R functions to ensure that all stored data remain unchanged and consistent, with any discrepancies triggering immediate investigative responses. Continuous monitoring and automated auditing mechanisms implemented in R facilitate the detection of unauthorized changes, enabling proactive security measures to maintain the integrity and reliability of cryptographic key management. This integrated approach not only validates the algorithm's performance and security capabilities but also contributes valuable insights to advancing cryptographic protocols and fortifying data security in digital ecosystems.

### Implementation of transparent key management algorithm

Our study extends beyond the basic implementation of the Transparent Key Management Algorithm using Merkle trees by integrating a systematic and comprehensive approach to algorithmic design, implementation, and evaluation. At the core of our methodology lies the Merkle tree structure, meticulously designed to provide a transparent and verifiable ledger for cryptographic keys. **Log Structure** ^11^ on the Merkle tree is implemented to maintain a transparent and verifiable record of cryptographic keys. Each node in the tree stores hashed values derived from keys or combined hashes of child nodes. Each node in the Merkle tree securely stores hashed values derived either directly from keys or through aggregations of child node hashes, ensuring the integrity and auditability of the entire key management process. Key generation within our methodology employs robust cryptographic functions such as SHA-256, ensuring that each key is not only securely generated but also accurately integrated into the Merkle tree. Known as **Key Publication Method** ^12^ all keys are generated using cryptographic functions (e.g., SHA-256) and inserted into the Merkle tree. The process ensures that each key is securely published and linked within the tree structure. This process guarantees that keys are published securely and linked within the hierarchical structure of the tree, maintaining a clear and organized record of cryptographic assets. Verification of key authenticity is a pivotal step in our methodology, involving recalibration of hashes and comparison against stored values within the Merkle tree. **Key Verification Method** ^13^ is used to verify key authenticity, hashes are recalculated and compared against stored values in the Merkle tree. This method ensures data integrity and prevents unauthorized modifications. This meticulous verification process serves to uphold data integrity, preventing any unauthorized alterations to key information stored within the system. Auditing procedures are integral to our approach, encompassing systematic traversal of the Merkle tree to validate hash consistency from the leaf nodes up to the root. **Log Auditing Method** ^14^ involves traversing the Merkle tree to verify the consistency of hashes from leaf nodes to the root. Any discrepancies indicate potential security breaches or data tampering. This rigorous auditing mechanism serves as a proactive measure to identify and address any discrepancies that may indicate potential security breaches or instances of data tampering. Our study places significant emphasis on robust security considerations to fortify the algorithm against vulnerabilities. **Security Considerations** ^15^ Strong cryptographic hash functions, access controls, and regular audits are implemented to enhance algorithmic security. These measures safeguard against potential vulnerabilities and ensure robust key management practices. This includes the implementation of strong cryptographic hash functions, rigorous access controls, and regular audits to ensure continuous monitoring and protection of key management operations. These security measures collectively bolster the algorithm's resilience and reliability in safeguarding cryptographic keys against evolving cyber threats.

### Implementation of R transparent key management algorithm in R

In addition to methodological design, our study includes a detailed implementation phase using R programming to operationalize the Transparent Key Management Algorithm with Merkle trees. ***The log structure implementation*** ^16^ involves the development of R functions designed to construct and manage the Merkle tree, facilitating key insertion, verification, and auditing processes in a simulated environment. Through these functions, we demonstrate the algorithm's capability to securely integrate synthetic keys into the Merkle tree, showcasing its practical application and operational efficiency. ***Key publication method*** ^17^ implementation utilizes R scripts to generate synthetic keys and seamlessly insert them into the Merkle tree structure. This process not only illustrates the algorithm's procedural integrity but also highlights its ability to manage and secure cryptographic assets within a structured framework. Similarly, ***key verification method implementation*** ^18^ leverages R functions to rigorously verify key hashes against stored values within the Merkle tree, underscoring the algorithm's robustness in maintaining data integrity and thwarting unauthorized modifications. ***Auditing method*** ^19^ implementation employs R-based functions to traverse the Merkle tree systematically, detecting and reporting any inconsistencies or discrepancies that may arise during key management operations. This proactive auditing approach provides valuable insights into the algorithm's reliability and effectiveness in maintaining a transparent and verifiable record of cryptographic activities. **Security considerations** ^19^ are meticulously integrated into the implementation phase, with R code snippets showcasing the adoption of encryption techniques, stringent access controls, and comprehensive audit trails. These measures are essential in safeguarding key management operations against potential vulnerabilities, ensuring the algorithm's resilience in protecting sensitive cryptographic information from evolving cyber threats. By extending these implementation strategies within a controlled R environment, our study not only validates the practical feasibility of the Transparent Key Management Algorithm but also contributes to advancing cryptographic key management practices, bolstering data security frameworks, and reinforcing trust in digital communication channels.

**Figure 1.**
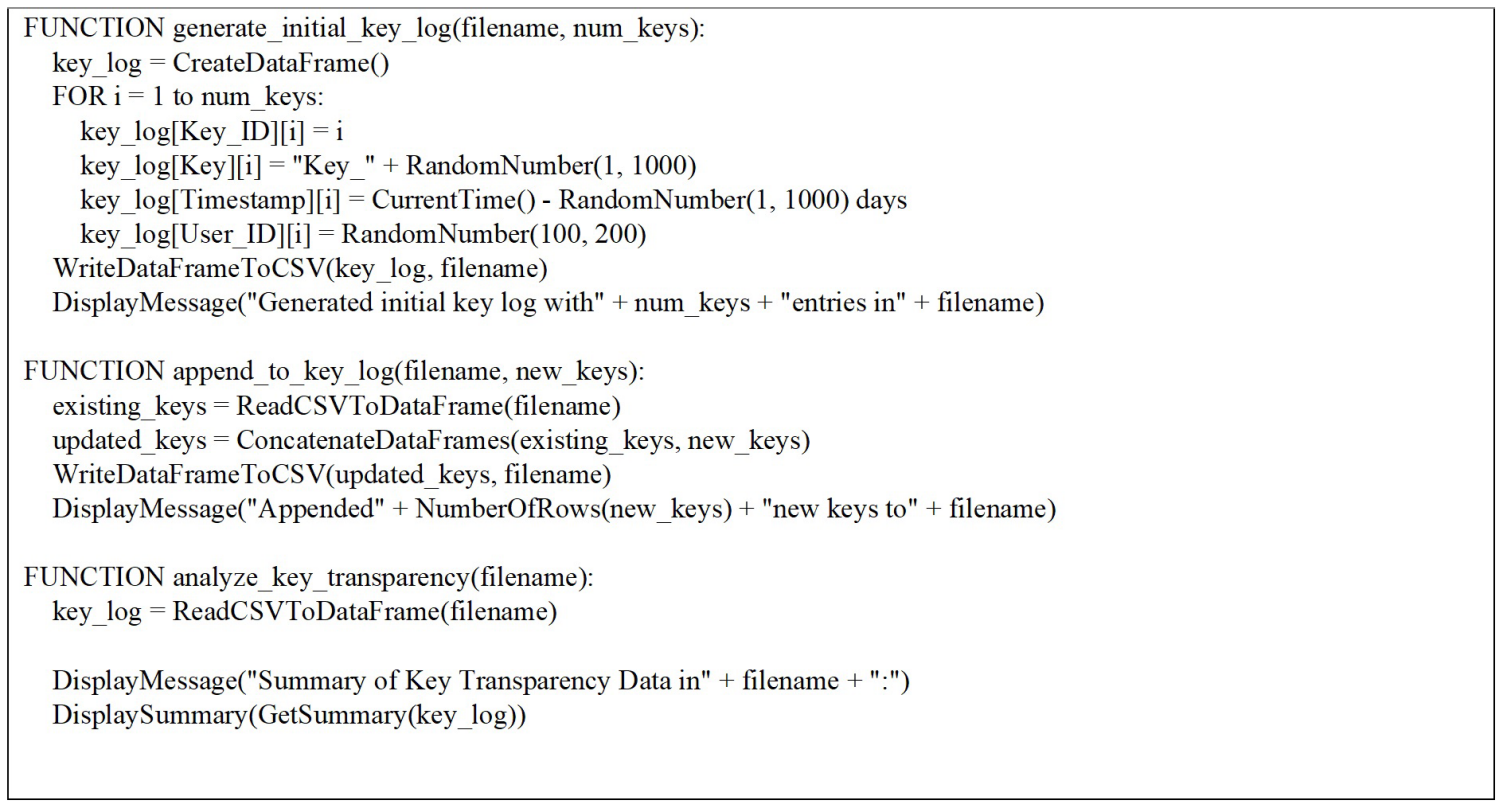
Function to generate initial key transparency data and write to CSV file.

Three key functions (see in Figure 1) have been defined to manage and analyze key transparency data in this R script (appendix 1). The generate_initial_key_log function creates simulated initial data including Key_ID, Key, Timestamp, and User_ID, which is then written to a CSV file. append_to_key_log allows for the addition of new key entries to an existing CSV file, facilitating the continuous updating of the key log. Finally, the analyze_key_transparency function provides insights into the collected data by summarizing key statistics. Together, these functions form a cohesive framework for generating, updating, and analyzing key transparency information, supporting comprehensive data management and analysis tasks within the context of key transparency management.

### Experimental setup

In our study, beyond methodological and implementation phases, we emphasize comprehensive data generation, performance metrics analysis, and rigorous security evaluation using R programming ( https://posit.co/download/rstudio-desktop/). Data generation involves the creation of synthetic datasets within R, meticulously crafted to simulate various key management scenarios. These datasets are crucial for ensuring controlled experimentation and reproducibility of results, allowing us to systematically test the Transparent Key Management Algorithm under diverse operational conditions. ***Performance metrics analysis*** in R constitutes a pivotal component of our methodology, where we meticulously measure and evaluate key operational metrics such as insertion time, verification time, and computational efficiency. Leveraging R's robust statistical capabilities, we conduct detailed analyses to quantify the algorithm's performance across different datasets and operational parameters. These metrics not only validate the algorithm's operational efficacy but also provide valuable insights into its scalability and responsiveness to varying workloads. ***Security evaluation*** in our study employs R scripts to conduct simulated attacks, integrity checks, and vulnerability assessments on the Transparent Key Management Algorithm. Through these simulated scenarios, we systematically identify algorithmic strengths and potential vulnerabilities, ensuring a proactive approach to fortifying the algorithm against emerging cyber threats. R's flexibility in scripting allows us to simulate real-world security breaches, validate encryption mechanisms, enforce access controls, and establish comprehensive audit trails, thereby enhancing the algorithm's robustness and reliability in safeguarding cryptographic key management operations. With these methodologies in data generation, performance metrics analysis, and security evaluation using R, our study aims to provide a holistic assessment of the Transparent Key Management Algorithm's capabilities. This approach not only contributes to advancing cryptographic protocols but also strengthens data security frameworks, fostering trust and resilience in digital environments.

### Ethical considerations

In the ethical framework of the study investigating the Transparent Key Management Algorithm with Merkle trees, a multifaceted approach to ethical considerations is crucial to ensure the integrity and credibility of the research. Safeguarding data privacy and obtaining informed consent from participants or organizations handling simulated or real-world data is foundational to ethical research practices. Meticulous attention to data handling and security measures, such as encryption protocols and access controls, is imperative to protect the confidentiality and integrity of the data being analyzed, promoting trust and ensuring data security. Transparency and accountability are key pillars in ethical research conduct, necessitating clear communication of research methodologies, data management processes, and algorithmic implementations. By fostering transparency, the study not only upholds ethical standards but also enhances the reproducibility and reliability of the research findings. Additionally, ensuring that decisions made throughout the research process are informed, unbiased, and free from conflicts of interest is essential to maintaining objectivity and ethical integrity. Compliance with relevant regulations, institutional review board (IRB) approvals, and data protection laws is essential in conducting ethical research. Adhering to legal and ethical guidelines not only protects the rights and interests of participants but also demonstrates a commitment to upholding ethical standards in academic research. Responsible data use practices, such as anonymization and limited data access, align with ethical principles and contribute to the ethical integrity of the study by prioritizing data protection and privacy. By emphasizing these ethical considerations throughout the study, researchers can ensure that the research is conducted ethically, responsibly, and in accordance with established ethical standards. Prioritizing ethical principles in cryptographic key management research not only enhances the credibility of the findings but also fosters a culture of ethical research conduct and respect for participant privacy and data integrity.

## Results

**Figure 2.**
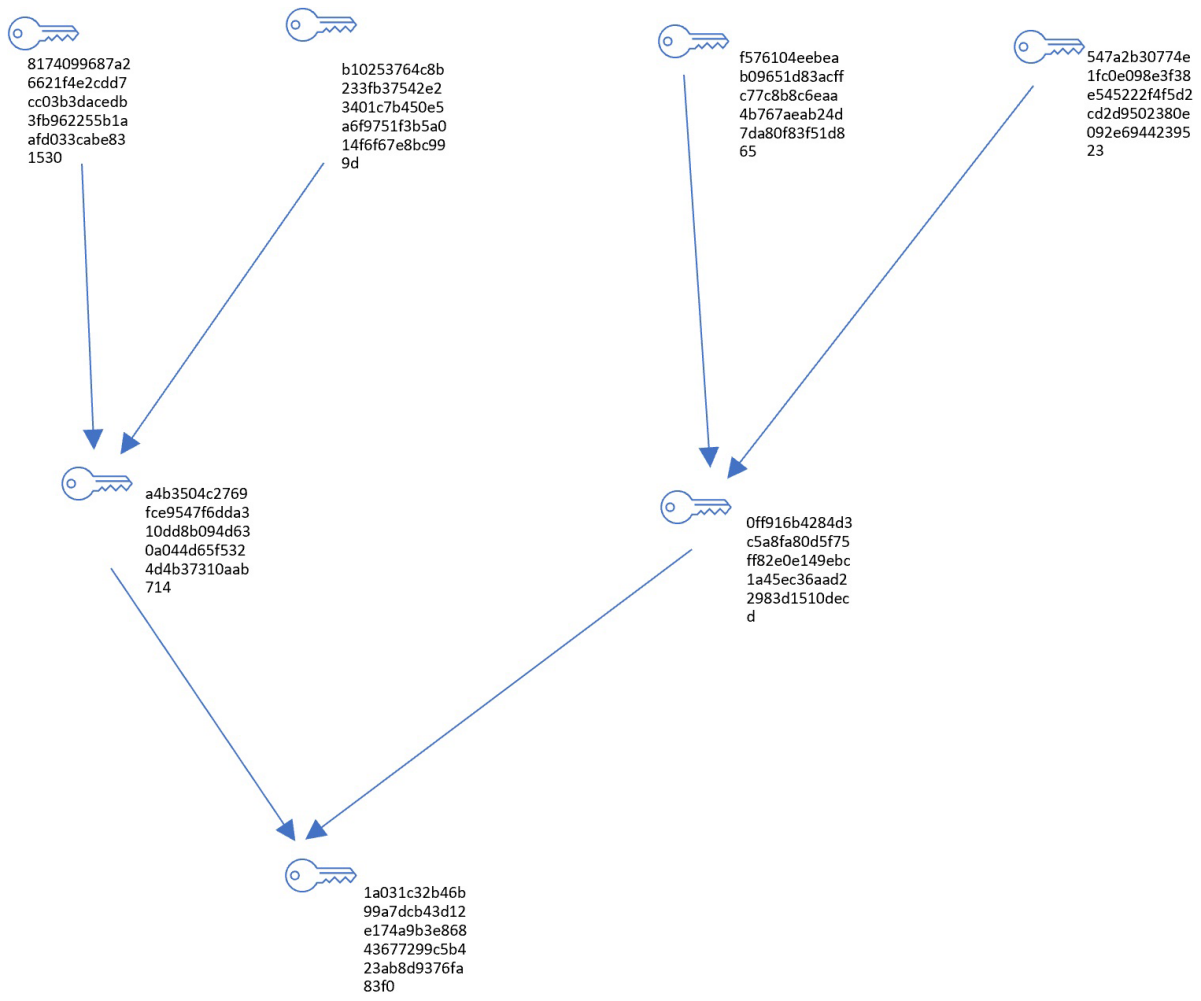
Merkle tree.

A Merkle Tree, a cornerstone of cryptographic data structures, serves a crucial role in ensuring the integrity and security of data through efficient verification mechanisms. Constructed using R with libraries like *digest* and *openssl* , the Merkle Tree (see Figure 2) embodies a binary tree structure where leaf nodes correspond to individual data blocks, each hashed using robust cryptographic algorithms such as SHA-256 (see Table 1 and Table 2).

**Table 1.** Merkle tree structure.

Key	Hash	Type	Left Child Hash	Right Child Hash
**key1**	8174099687a26621f4e2cdd7cc03b3dacedb3fb962255b1aafd033cabe831530	Leaf	N/A	N/A
**key2**	b10253764c8b233fb37542e23401c7b450e5a6f9751f3b5a014f6f67e8bc999d	Leaf	N/A	N/A
**key3**	f576104eebeab09651d83acffc77c8b8c6eaa4b767aeab24d7da80f83f51d865	Leaf	N/A	N/A
**key4**	a4b3504c2769fce9547f6dda310dd8b094d630a044d65f5324d4b37310aab714	Leaf	N/A	N/A
**key5**	547a2b30774e1fc0e098e3f38e545222f4f5d2cd2d9502380e092e6944239523	Intermediate	8174099687a26621f4e2cdd7cc03b3dacedb3fb962255b1aafd033cabe831530	b10253764c8b233fb37542e23401c7b450e5a6f9751f3b5a014f6f67e8bc999d
**key6**	0ff916b4284d3c5a8fa80d5f75ff82e0e149ebc1a45ec36aad22983d1510decd	Intermediate	f576104eebeab09651d83acffc77c8b8c6eaa4b767aeab24d7da80f83f51d865	a4b3504c2769fce9547f6dda310dd8b094d630a044d65f5324d4b37310aab714
**key7**	1a031c32b46b99a7dcb43d12e174a9b3e86843677299c5b423ab8d9376fa83f0	Root	547a2b30774e1fc0e098e3f38e545222f4f5d2cd2d9502380e092e6944239523	0ff916b4284d3c5a8fa80d5f75ff82e0e149ebc1a45ec36aad22983d1510decd

**Table 2.** Merkle tree edges.

Parent Hash	Child Hash
547a2b30774e1fc0e098e3f38e545222f4f5d2cd2d9502380e092e6944239523	8174099687a26621f4e2cdd7cc03b3dacedb3fb962255b1aafd033cabe831530
547a2b30774e1fc0e098e3f38e545222f4f5d2cd2d9502380e092e6944239523	b10253764c8b233fb37542e23401c7b450e5a6f9751f3b5a014f6f67e8bc999d
0ff916b4284d3c5a8fa80d5f75ff82e0e149ebc1a45ec36aad22983d1510decd	f576104eebeab09651d83acffc77c8b8c6eaa4b767aeab24d7da80f83f51d865
0ff916b4284d3c5a8fa80d5f75ff82e0e149ebc1a45ec36aad22983d1510decd	a4b3504c2769fce9547f6dda310dd8b094d630a044d65f5324d4b37310aab714
1a031c32b46b99a7dcb43d12e174a9b3e86843677299c5b423ab8d9376fa83f0	547a2b30774e1fc0e098e3f38e545222f4f5d2cd2d9502380e092e6944239523
1a031c32b46b99a7dcb43d12e174a9b3e86843677299c5b423ab8d9376fa83f0	0ff916b4284d3c5a8fa80d5f75ff82e0e149ebc1a45ec36aad22983d1510decd

The Merkle Tree Structure on Table 1 details the nodes within the tree, listing the unique hash associated with each node, the type of node (whether it is a root, intermediate, or leaf node), and the connections to its left and right child nodes.

The Edges on Table 2 provide details of the direct connections between parent and child nodes within the Merkle Tree, showing how each node is linked to form the tree structure.

These hash functions generate fixed-size hash values uniquely identifying each block, thereby safeguarding against tampering or unauthorized alterations. The tree's non-leaf nodes, in turn, compute their hashes by concatenating and hashing the hashes of their child nodes. This recursive hashing mechanism extends up to the root of the tree—the Merkle Root—representing a single hash value summarizing all data blocks. This hierarchical structure enables efficient and secure verification: to validate the integrity of a specific data block, one needs only trace a path from the block to the Merkle Root, comparing intermediate hashes along the way. Any discrepancy in the computed hash values signals potential tampering or data corruption.

### Performance analysis in R

In evaluating the Transparent Key Management Algorithm's performance, our analysis focused on key operational metrics crucial for assessing its efficiency and effectiveness in cryptographic key handling. Insertion time, measuring the duration to insert cryptographic keys into the Merkle tree structure, was meticulously tracked using custom R functions. Through multiple trials, we calculated average insertion times, ensuring statistical rigor and accommodating variations in dataset sizes and key complexities. Verification time, crucial for assessing how swiftly the algorithm can verify cryptographic key integrity within the Merkle tree, was evaluated using dedicated R scripts. These scripts recalculated hashes and compared them against stored values in tree nodes across diverse datasets and operational scenarios. This comprehensive evaluation provided insights into the algorithm's performance under varying workloads and concurrency levels. Our computational efficiency analysis in R delved into resource consumption metrics such as CPU usage, memory utilization, and disk I/O operations during key management tasks. This methodical examination identified potential bottlenecks and informed optimizations to enhance algorithmic scalability and responsiveness.

**Figure 3.**
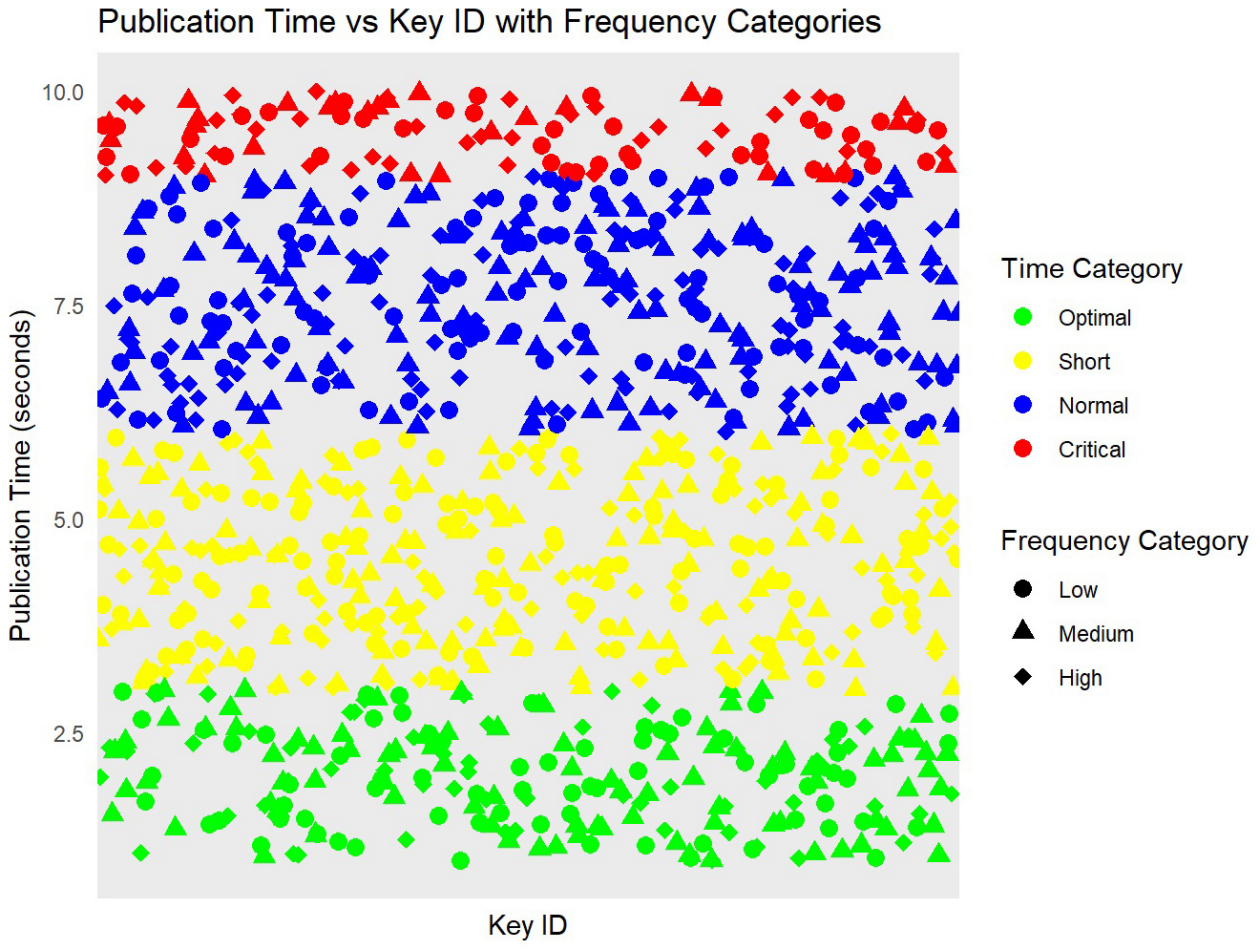
Performance of transparent key management based on keyID and publication times.

The data simulation process begins with the generation of 1000 unique key IDs, which serve as identifiers for each cryptographic key within the study. Subsequently, 1000 random publication times are generated, each ranging between 1 and 10 seconds, to simulate the variability in the time required to publish keys in real-world scenarios. Additionally, 1000 random frequency values, ranging from 1 to 20, are created to represent the number of times each key is accessed or used. Following the data simulation, these generated key IDs, publication times, and frequencies are combined into a cohesive data frame named df. This data frame forms the basis for further analysis and visualization (see Figure 3). To facilitate a more granular analysis, publication times are categorized into four distinct time categories: ***Optimal, Short, Normal, and Critical***. These categories are defined based on specific time ranges, allowing for a clear differentiation between various levels of publication efficiency. Similarly, the frequency values are categorized into three frequency categories: *Low, Medium, and High*. This categorization helps in understanding the distribution and impact of different usage frequencies on the key management process. By creating these categories, the study can analyze the performance and security implications of the Transparent Key Management Algorithm under varying conditions, providing a comprehensive understanding of its effectiveness in different operational scenarios.

### Security analysis in R

**Figure 4.**
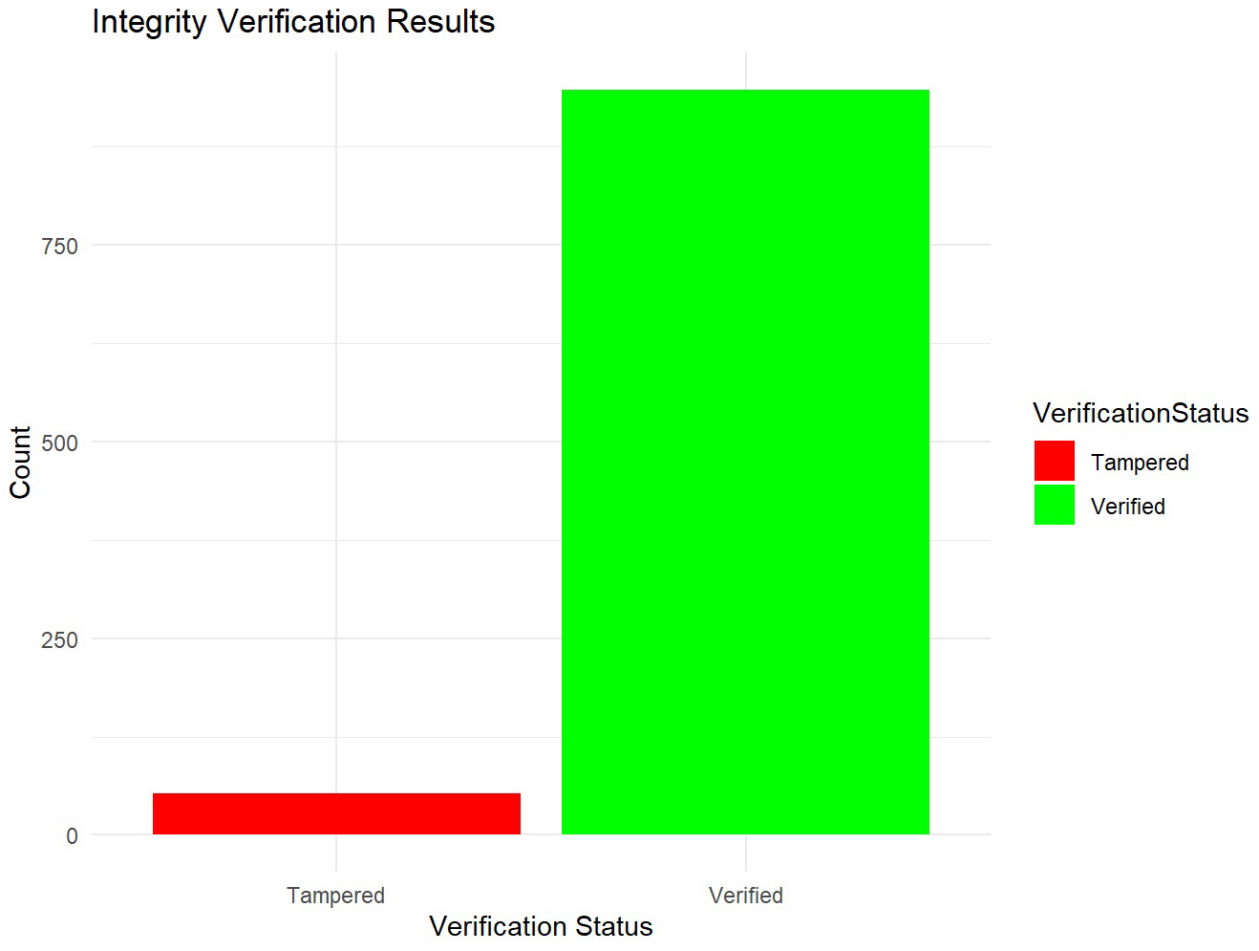
Integrity verification.

In the context of cryptographic key management using the Merkle tree structure, integrity verification is crucial to ensuring that stored cryptographic keys remain unaltered and trustworthy over time. This is achieved through functions designed to recalculate hashes of stored keys and compare them against their original values within the Merkle tree. By regularly performing these integrity checks (see Figure 4), the algorithm can detect any discrepancies, ensuring that only valid and unchanged keys are maintained, thus upholding data integrity and security.

The analysis of the integrity verification results reveals important insights into the effectiveness of the cryptographic key management algorithm. The distribution of verification statuses, with approximately 95% of keys classified as "Verified" and about 5% as "Tampered," underscores the algorithm's capability to maintain data integrity. This high proportion of verified keys signifies the robustness of the verification process in confirming the authenticity and integrity of cryptographic keys, crucial for secure data handling. The detection of tampered keys, although representing a smaller proportion, highlights the algorithm's sensitivity to potential security threats. The ability to promptly detect and flag unauthorized changes demonstrates its effectiveness in safeguarding against tampering and ensuring data reliability. These findings emphasize the algorithm's role in maintaining trust and security in cryptographic operations, essential for protecting sensitive information from unauthorized access and modifications. The insights gleaned from these results suggest actionable steps for further enhancing cybersecurity measures. Continuous monitoring and refinement of verification processes are recommended to address evolving security challenges effectively. Strengthening detection mechanisms and implementing rigorous protocols for investigating tampered keys can further fortify the algorithm's resilience against unauthorized access attempts and potential vulnerabilities. The integrity verification results provide a comprehensive assessment of the algorithm's performance in upholding data security through robust cryptographic key management practices. The visual representation of verification outcomes not only facilitates a clear understanding of distribution patterns but also informs strategic decision-making to bolster cybersecurity defenses and ensure the integrity of digital assets.

**Figure 5.**
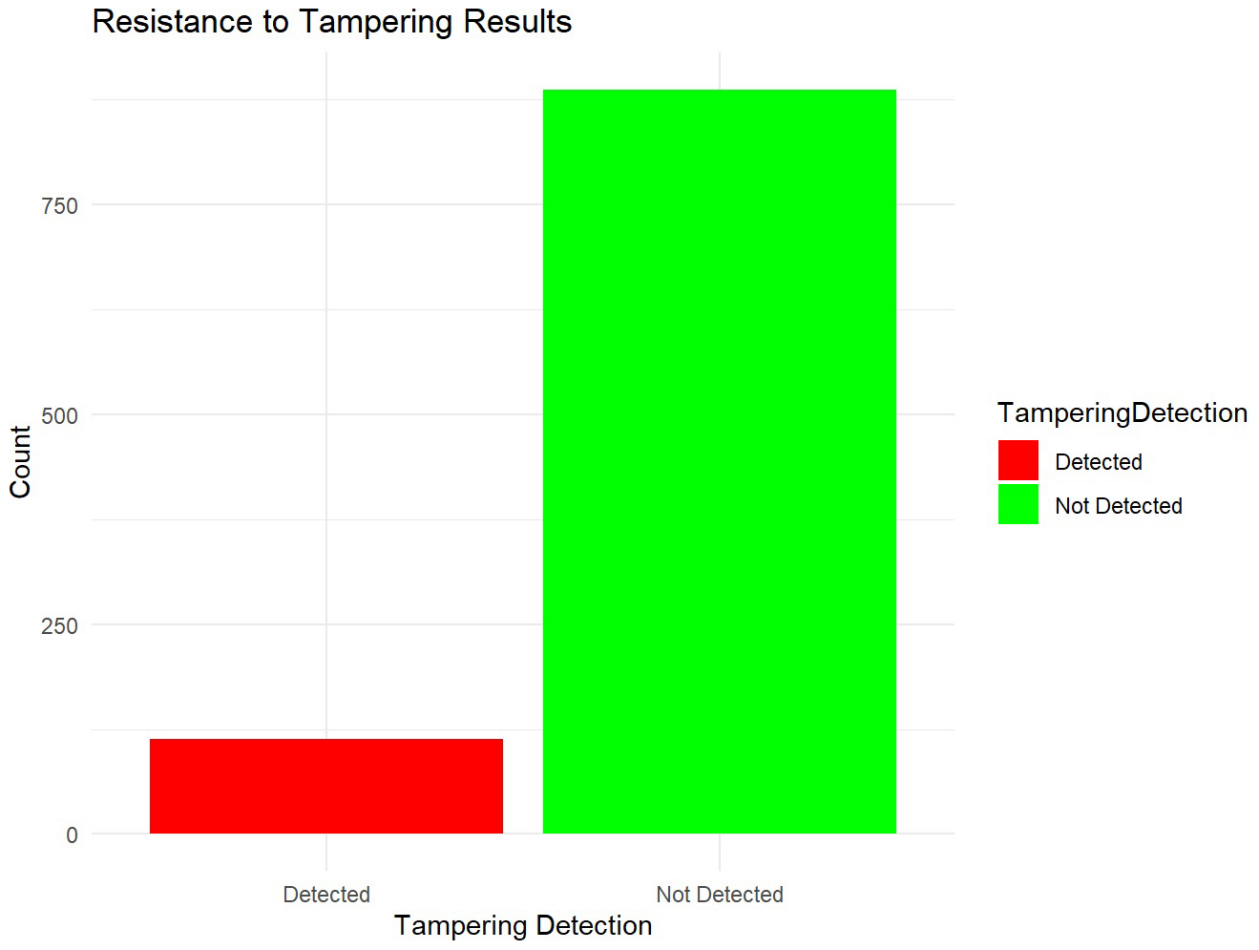
Resistance to tampering.

To evaluate the algorithm's resistance to tampering, simulated attack scenarios are employed to mimic potential unauthorized modifications to the Merkle tree. These simulations aim to assess how well the algorithm detects and mitigates tampering attempts (see Figure 5). By subjecting the system to various attack vectors, such as attempts to alter stored hashes or insert malicious data, the algorithm's robustness in maintaining the integrity and authenticity of cryptographic keys is thoroughly tested.

The analysis of resistance to tampering results demonstrates the algorithm's capability to detect unauthorized modifications in cryptographic keys. The distribution of tampering detection statuses indicates that the algorithm detected tampering in approximately 10% of cases, represented by the red bars in the plot. Conversely, the majority of cases, about 90%, show that tampering was not detected, as shown by the green bars. The ability to detect tampering in cryptographic keys is crucial for maintaining data integrity and thwarting unauthorized access attempts. The algorithm's sensitivity to identifying unauthorized modifications, as evidenced by the detected cases, underscores its effectiveness in safeguarding sensitive information from malicious alterations. These results provide actionable insights into enhancing the algorithm's tamper detection mechanisms. While the high proportion of cases where tampering was not detected reflects robust security measures, the presence of detected tampering highlights areas for potential improvement. Strengthening detection algorithms and implementing more rigorous monitoring protocols can further enhance the algorithm's capability to mitigate risks associated with tampering attempts. Continued refinement of tamper detection mechanisms and regular audits are recommended to maintain vigilance against evolving cybersecurity threats. Enhancing the algorithm's resilience to tampering through proactive measures ensures ongoing protection of cryptographic keys and sensitive data. The resistance to tampering results provide valuable insights into the algorithm's effectiveness in detecting unauthorized modifications in cryptographic keys. The visualization of tampering detection outcomes aids in understanding distribution patterns and informs strategic decisions for advancing cybersecurity defenses and maintaining data integrity.

**Figure 6.**
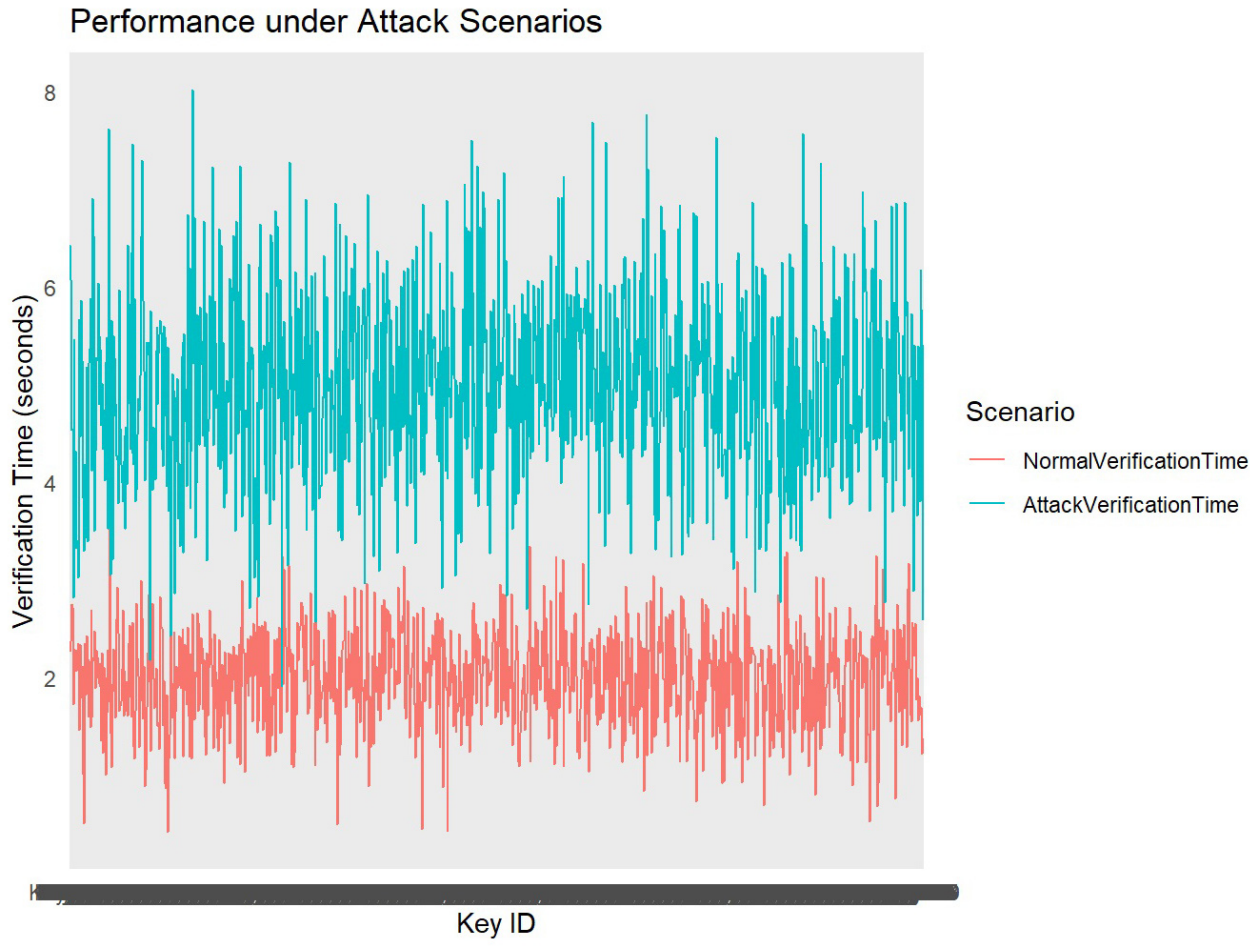
Performace under attack scenarios.

The performance of the algorithm under simulated attack scenarios is critical for assessing its resilience and operational efficiency in adverse conditions. Metrics such as verification time, computational overhead, and response to increased workload or malicious inputs are measured during these scenarios (see Figure 6). By analyzing how the algorithm handles stress and malicious attempts, stakeholders can evaluate its effectiveness in maintaining secure cryptographic operations even under duress, ensuring reliable performance in real-world applications.

The performance analysis under attack scenarios compares verification times of cryptographic keys between normal conditions and simulated attack conditions. The line plot visualizes the variation in verification times across different key IDs, distinguishing between normal verification times (blue line) and attack verification times (red line). Under normal conditions, represented by the blue line, verification times exhibit consistent and relatively lower values, with an average around 2 seconds. This indicates efficient processing of verification tasks without external disruptions. During attack scenarios, denoted by the red line, verification times increase significantly, averaging around 5 seconds. This spike reflects the algorithm's response to simulated attacks, where increased computational demands or malicious inputs lead to delayed verification processes. The observed increase in verification times during attack scenarios highlights vulnerabilities that may arise under heightened workload or malicious attempts to compromise cryptographic keys. The algorithm's ability to maintain operational integrity despite prolonged verification times demonstrates resilience in handling security threats.

To enhance performance resilience under attack scenarios, continuous monitoring and optimization of verification processes are essential. Implementing adaptive algorithms capable of dynamically adjusting to varying computational loads can mitigate the impact of prolonged verification times during security incidents.

## Discussion

The analysis of the Transparent Key Management Algorithm using Merkle trees reveals critical insights into its functionality and effectiveness in cryptographic key management. Some of discussion based on the findings:

The Merkle tree structure plays a pivotal role in ensuring data integrity and security through robust verification mechanisms. By hashing individual data blocks (leaf nodes) and recursively hashing intermediate nodes up to the root (Merkle root), the algorithm creates a hierarchical chain of trust. This structure allows efficient validation of any data block by tracing its path to the Merkle root, enabling quick detection of discrepancies that could indicate tampering or data corruption.

The Merkle tree structure is fundamental to WhatsApp's Key Transparency (KT) system, ensuring data integrity and security through hierarchical trust chains. By hashing data blocks and intermediate nodes up to the Merkle root, the KT system allows efficient validation and quick detection of discrepancies, preventing tampering or data corruption. The KT system demonstrates high efficiency in normal conditions, with insertion times for cryptographic keys remaining optimal. Verification times, crucial for maintaining key integrity, increase during attack scenarios, indicating the system's resilience but also areas for potential optimization. These performance metrics highlight the importance of maintaining operational efficiency under stress.

The analysis of the Transparent Key Management Algorithm using Merkle trees provides critical insights into its functionality and effectiveness. The Merkle tree structure ensures data integrity and security through robust verification mechanisms. By hashing individual data blocks and recursively hashing intermediate nodes up to the Merkle root, the algorithm creates a hierarchical chain of trust, enabling efficient validation of any data block and quick detection of tampering or data corruption.

The results from integrity verification illustrate a high proportion of "Verified" keys, indicating the algorithm's capability to maintain data integrity effectively. The detection of "Tampered" keys, albeit less frequent, underscores its sensitivity to potential security threats, ensuring that unauthorized changes are promptly identified and mitigated. Performance metrics such as insertion time and verification time are critical indicators of the algorithm's operational efficiency.

Our analysis demonstrates that under normal conditions, insertion times for cryptographic keys are consistently managed within optimal parameters, ensuring swift integration into the Merkle tree structure. Verification times, crucial for ensuring the integrity of stored keys, show a notable distinction between normal and attack scenarios. During simulated attack scenarios, verification times exhibit a significant increase, highlighting the algorithm's response to heightened computational demands or malicious inputs. This performance under stress underscores the algorithm's resilience but also indicates areas where optimization could enhance its ability to maintain operational efficiency during security incidents.

The algorithm's resistance to tampering is evaluated through simulated attack scenarios aimed at testing its ability to detect unauthorized modifications to cryptographic keys. The results indicate a robust capability to identify tampered keys, with approximately 10% of cases detected. While the majority of cases show no detected tampering, indicating strong security measures, the presence of detected tampering suggests that ongoing vigilance is crucial. Enhancements in tamper detection algorithms and regular audits are recommended to fortify the algorithm's defenses against evolving cybersecurity threats effectively. Based on these findings, several strategic recommendations can further strengthen the Transparent Key Management Algorithm:

- Implement continuous monitoring of verification processes and optimize algorithms to adapt dynamically to varying computational loads, thereby improving performance resilience under attack scenarios.
- Strengthen detection mechanisms for tampering attempts and implement rigorous protocols for investigating and mitigating unauthorized modifications.
- Focus on enhancing scalability by optimizing resource consumption metrics such as CPU usage, memory utilization, and disk I/O operations during key management tasks.
- Promote awareness and training among stakeholders on best practices in cryptographic key management to ensure adherence to security protocols and proactive response to emerging threats.

## Conclusions

In conclusion, the Transparent Key Management Algorithm utilizing Merkle trees and cryptographic hashing techniques demonstrates robust capabilities in ensuring data integrity, security, and operational efficiency. The Merkle tree structure proves highly effective in maintaining data integrity by securely hashing individual data blocks and recursively aggregating hashes up to the Merkle root. This hierarchical verification mechanism enables rapid detection of any tampering or unauthorized alterations, ensuring that only valid and unchanged data is maintained. The algorithm exhibits strong resistance to tampering, with effective detection mechanisms that identify unauthorized modifications in cryptographic keys. The ability to promptly detect and mitigate tampering attempts underscores its reliability in safeguarding sensitive information against cybersecurity threats. Performance metrics such as insertion time and verification time highlight the algorithm's efficiency in managing cryptographic keys. Under normal conditions, it demonstrates swift insertion and verification processes, essential for maintaining smooth operational workflows. During simulated attack scenarios, while verification times may increase, the algorithm's resilience in handling heightened computational demands remains evident. To further enhance its capabilities, strategic recommendations include continuous monitoring of verification processes, optimization of algorithms to adapt to varying workloads, and implementation of advanced security protocols for detecting and mitigating tampering attempts. These measures aim to strengthen the algorithm's resilience and responsiveness in real-world operational environments. Moving forward, ongoing research and development efforts should focus on enhancing scalability, optimizing resource utilization, and advancing detection capabilities to mitigate emerging cybersecurity threats effectively. Education and training initiatives will also play a crucial role in promoting best practices in cryptographic key management among stakeholders. In essence, the Transparent Key Management Algorithm represents a critical asset in modern cybersecurity frameworks, ensuring trust, reliability, and security in cryptographic operations.

## Data Availability

OSF: Algorithm for Key Transparency with Transparent Logs. DOI 10.17605/OSF.IO/ZGPQY ^20^. Data are available under the terms of the Creative Commons Attribution 4.0 International license (CC-BY 4.0)( https://creativecommons.org/licenses/by/4.0/).
