# Supplementary material for: Algorithm for Key Transparency with Transparent Logs
Source: Open Res Eur. 2024 Oct 2;4:163. Originally published 2024 Aug 5. [Version 2] doi: 10.12688/openreseurope.18200.2 (PMC11585852; doi:10.12688/openreseurope.18200.2)
Supplement: Peer review history

# Reviewer report: Reviewer response for version 2

**DOI:** 10.21956/openreseurope.19938.r46939

**Published:** 2024-11-23

**Authors:** K Kuppusamy

**Affiliations:** 1 Dept. of Computational Logistics, Alagappa University, Karaikudi, India

Article title: Algorithm for Key Transparency with Transparent Logs

 Prof.K.Kuppusamy

 Dept. of Computational Logistics, Alagappa University, Karaikudi, Tamilnadu, India.

 The manuscript (revised) entitled "Algorithm for key transparency with transparent logs" has been reviewed. The comments/findings are given below:

 •    This article presents a novel approach for cryptographic key management by devising a transparent key management algorithm with Merkle tree structure and hashing techniques to ensure data integrity, security, and cryptographic operational efficiency and to address transparency and robustness in key handling.  

 •    The manuscript is clearly written, well-structured and ensures academic quality.

 •    Technical details and importance of transparent key management have been described in detail in the manuscript.

 •    The methodology for devising the key transparency algorithm with Merkle tree structure has been explained clearly.  This algorithm provides a structured framework for cryptographic key management and to address transparency and verifiability challenges. Besides, the implementation of transparent key management algorithm with step-by-step procedure is also been explained in detail. 

 •    The key management activities such as verifiable key directory creation, initial key logs and keys generation, inserting keys and analyzing key transparency are performed using built in functional modules in R programming environment and presented clearly in the manuscript.

 •    Performance of the transparent key management algorithm is evaluated in terms of the metrics like key insertion time, verification time and computational efficiency. Though the experimental results are shown through graphical representation, it may improve the clarity if the performance metrics are given in tabular form.

 •    It is appreciated that the Security analysis of the proposed key transparent algorithm is performed through simulated attacks in R programming environment by observing the algorithms resilience against tampering, integrity verification and detection of unauthorized modifications. 

 •    Results and discussion on experimental and analysis of transparent key management algorithm are described in detail.

 •    Summary and significant key points of research contribution are given in the conclusion part politely.

    Based on the review, this research article is recommended for Indexing

Is the study design appropriate and does the work have academic merit?

Yes

Is the work clearly and accurately presented and does it cite the current literature?

Yes

If applicable, is the statistical analysis and its interpretation appropriate?

Not applicable

Are all the source data underlying the results available to ensure full reproducibility?

Partly

Are the conclusions drawn adequately supported by the results?

Yes

Are sufficient details of methods and analysis provided to allow replication by others?

Yes

Reviewer Expertise:

Cryptography, Network Security

I confirm that I have read this submission and believe that I have an appropriate level of expertise to confirm that it is of an acceptable scientific standard.

# Reviewer report: Reviewer response for version 2

**DOI:** 10.21956/openreseurope.19938.r44888

**Published:** 2024-10-18

**Authors:** Frimpong Twum, John Kwao Dawson

**Affiliations:** 1 Kwame Nkrumah University of Science & Technology, Kumasi, Ghana; 2 Computer Science, Sunyani Technical University (Ringgold ID: 505162), Sunyani, Brong Ahafo Region, Ghana

Algorithm for Key Transparency with Transparent Logs

 The manuscript dubbed “Algorithm for Key Transparency with Transparent Logs” has been reviewed and the following concerns have been raised.

1. The source of the first paragraph in the manuscript should be cited
2. The structure of the manuscript is making it difficult to appreciate
3. There is no chronology in the manuscript. There are so many miss ups in the manuscript leading to addition of irrelevant materials.
4. The authors should explain the importance of the repetition of Literature Review and Related work. How are they related and different from each other
5. The methodology employed in the manuscript is not well elaborated making it difficult for reproducibility. For example, the authors have stated in their work that” Keys are generated and inserted into the Merkel tree using specialized function. Which function? They should be specific in the type of hash algorithm used and justify the reason for the selection of such function.
6. Also from the step-by-step procedure in the manuscript, the proposed method is able to resist cyber threats. How?
7. What is the difference between the two different headings “Implementing R on TKMA and Performance analysis and implementation of TKMA in R. This makes the manuscript very confusing.
8. The authors have indicated that their framework performed 95% approximately of keys classified as “Verified” and robustness of the verification process in confirming the authenticity and integrity of cryptographic keys. Against which of the related works did the authors compared their work with to make this generalization.
9. The Merkel tree is not correct because which of the nodes is the root node. It is difficult to identify the dependents to construct the root nodes.
10. The authors should prove the robustness of their proposed algorithm mathematically against cyber-attacks as stated in the manuscript.

In summary the manuscript is not well developed and is difficult to assimilate.

Is the study design appropriate and does the work have academic merit?

No

Is the work clearly and accurately presented and does it cite the current literature?

Partly

If applicable, is the statistical analysis and its interpretation appropriate?

Not applicable

Are all the source data underlying the results available to ensure full reproducibility?

No

Are the conclusions drawn adequately supported by the results?

No

Are sufficient details of methods and analysis provided to allow replication by others?

No

Reviewer Expertise:

Computer Security

We confirm that we have read this submission and believe that we have an appropriate level of expertise to state that we do not consider it to be of an acceptable scientific standard, for reasons outlined above.

# Reviewer report: Reviewer response for version 2

**DOI:** 10.21956/openreseurope.19938.r44814

**Published:** 2024-10-11

**Authors:** Muhammad Azhar Mushtaq

**Affiliations:** 1 University of Sargodha, Sargodha, Punjab, Pakistan

Paper has been revised by the authors and I have no further inquiries. I recommend the paper to be indexed.

Is the study design appropriate and does the work have academic merit?

Partly

Is the work clearly and accurately presented and does it cite the current literature?

Partly

If applicable, is the statistical analysis and its interpretation appropriate?

Partly

Are all the source data underlying the results available to ensure full reproducibility?

Partly

Are the conclusions drawn adequately supported by the results?

Partly

Are sufficient details of methods and analysis provided to allow replication by others?

Partly

Reviewer Expertise:

Crptography, CyberSecurity, Network Security, BlockChain, Internet of Things

I confirm that I have read this submission and believe that I have an appropriate level of expertise to confirm that it is of an acceptable scientific standard.

# Reviewer report: Reviewer response for version 1

**DOI:** 10.21956/openreseurope.19670.r42868

**Published:** 2024-08-22

**Authors:** Muhammad Azhar Mushtaq

**Affiliations:** 1 University of Sargodha, Sargodha, Punjab, Pakistan

1. The clarity and structure of the paper could be improved to enhance its academic point of view and readability.

 2. The research objectives could benefit from further clarification, and a stronger connection between the results with the objectives would strengthen the overall argument.

 3. Additional details on the implementation process would be valuable, although the titles have implementation inside. 

 4. Some sections of the article include fundamental information that may not contribute significantly to the researchers.

 5. The literature review section would benefit from a more comprehensive examination of past research papers, including a discussion of their drawbacks to better contextualize the current research paper.

 6. Figure 1 and Figure 2 require more clarification in the text, currently it is not effectively conveying the intended information.

 7. Further discussion on newer types of attacks should also be considered in the experimental procedure.

Is the study design appropriate and does the work have academic merit?

Partly

Is the work clearly and accurately presented and does it cite the current literature?

Partly

If applicable, is the statistical analysis and its interpretation appropriate?

Partly

Are all the source data underlying the results available to ensure full reproducibility?

Partly

Are the conclusions drawn adequately supported by the results?

Partly

Are sufficient details of methods and analysis provided to allow replication by others?

Partly

Reviewer Expertise:

Crptography, CyberSecurity, Network Security, BlockChain, Internet of Things

I confirm that I have read this submission and believe that I have an appropriate level of expertise to confirm that it is of an acceptable scientific standard, however I have significant reservations, as outlined above.

# Reviewer report: Reviewer response for version 1

**DOI:** 10.21956/openreseurope.19670.r42871

**Published:** 2024-08-16

**Authors:** Sedat Akleylek

**Affiliations:** 1 University of Tartu, Tartu, Estonia

In this paper, the authors provide some experimental results on the key transparency. 

**I have the following comments:**

 1. The organization is hard to follow. 

 2. Implementation details are not given although the titles have implementation inside.

 3. The manuscript contains several instances of repetition that could be streamlined to improve readability.

 4. Certain sections of the article present information that appears to be quite basic and might benefit from further development or elaboration. Technical details and importance of transparent key management are not discussed in detail. 

 5. Literature review does not serve the current state of the art.

 6. Figure 1 and Figure 2 need clarification, as their current presentation may not effectively convey the intended information.

 7. The purpose of Tables 1 and 2 is unclear; providing additional context or justification for their inclusion would be helpful.

 8. Discussion should be expanded by considering the real-world attacks.

Is the study design appropriate and does the work have academic merit?

Partly

Is the work clearly and accurately presented and does it cite the current literature?

Partly

If applicable, is the statistical analysis and its interpretation appropriate?

Partly

Are all the source data underlying the results available to ensure full reproducibility?

Partly

Are the conclusions drawn adequately supported by the results?

Partly

Are sufficient details of methods and analysis provided to allow replication by others?

Partly

Reviewer Expertise:

Cryptography, cyber security.

I confirm that I have read this submission and believe that I have an appropriate level of expertise to confirm that it is of an acceptable scientific standard, however I have significant reservations, as outlined above.
